# Editorial: The RNA revolution and cancer

**DOI:** 10.3389/fendo.2024.1422599

**Published:** 2024-05-20

**Authors:** Zodwa Dlamini, Michael R. Ladomery, Abdullah Kahraman

**Affiliations:** ^1^ SAMRC Precision Oncology Research Unit (PORU), DSI/NRF SARChI Chair in Precision Oncology and Cancer Prevention (POCP), University of Pretoria, Pretoria, South Africa; ^2^ School of Applied Sciences, University of the West of England, Bristol, United Kingdom; ^3^ School of Life Sciences, University of Applied Sciences and Arts Northwestern Switzerland, Muttenz, Switzerland; ^4^ Swiss Institute of Bioinformatics, Lausanne, Switzerland

**Keywords:** RNA biology, cancer, endocrine-related cancers, alternative splicing, noncoding RNAs, microRNAs, long noncoding RNAs, circular RNAs

## Abstract

RNA biology has revolutionized cancer understanding and treatment, especially in endocrine-related malignancies. This editorial highlights RNA's crucial role in cancer progression, emphasizing its influence on tumor heterogeneity and behavior. Processes like alternative splicing and noncoding RNA regulation shape cancer biology, with microRNAs, long noncoding RNAs, and circular RNAs orchestrating gene expression dynamics. Aberrant RNA signatures hold promise as diagnostic and prognostic biomarkers in endocrine-related cancers. Recent findings, such as aberrant PI3Kδ splice isoforms and epithelial-mesenchymal transition-related lncRNA signatures, unveil potential therapeutic targets for personalized treatments. Insights into m6A-associated lncRNA prognostic models and the function of lncRNA LINC00659 in gastric cancer represents ongoing research in this field. As understanding of RNA's role in cancer expands, personalized therapies offer transformative potential in managing endocrine-related malignancies. This signifies a significant stride towards precision oncology, fostering innovation for more effective cancer care.

## Introduction

**Figure 1:**
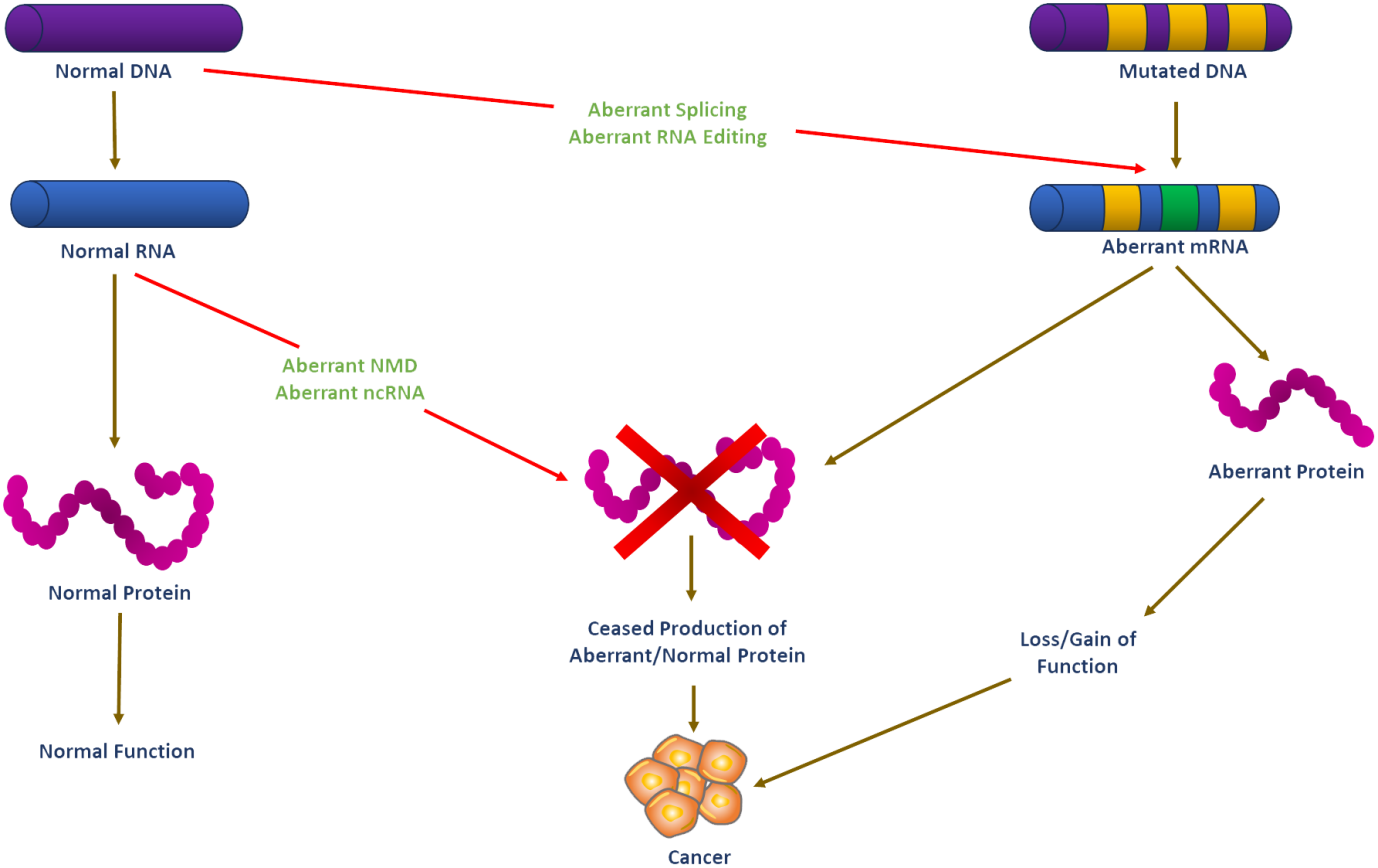
Schematic illustration of RNA-based systems and their putative regulatory functions in cancer development. Carcinogenesis is believed to be caused by DNA mutations in oncogene and tumor suppressor genes. Mutated mRNA translation can result in functionally altered proteins that may lead to cell transformation. RNA-based processes such as RNA editing, alternative splicing, ncRNA, and NMD may theoretically regulate the expression of these altered transcripts, but they are unlikely to prevent or stop cell transformation. However, functional dysregulation of these RNA-based systems may affect the expression of non-mutated genes. Such abnormal functioning of RNA-based systems may be the first step in the process of carcinogenesis before the emergence of mutations in DNA may eventually ‘fix’ the cell transformation process. DNA, Deoxyribonucleic acid; RNA, Ribonucleic acid; ncRNA, non-coding RNA; NMD, nonsense-mediated decay.

Over the last decades, molecular biology has seen remarkable discoveries that have increased our understanding of the molecular heterogeneity of cancer, including endocrine-related malignancies. A critical area that has seen significant development is RNA biology, which has been marked by a major increase in research articles covering its various facets. This growth in RNA biology literature has provided insights into the direct roles of RNA in cellular functions, illuminating the functional flexibility and structural intricacy of RNA molecules. Furthermore, there has been extensive development in understanding how proteins recognize and bind RNA sequences and structures (1). Also, it is now apparent that RNA-centered processes regulate gene expression in multiple ways, including co-transcriptional pre-mRNA processing, alternative splicing, mRNA translation, localization, stability, and editing (2, 3). The advent of regulatory noncoding RNAs, such as microRNAs (miRNAs), long noncoding RNAs (lncRNAs), and circular RNAs (circRNAs), has further added another layer of complexity to our understanding of gene regulation (4, 5). Aberrantly spliced mRNA isoforms and dysregulated splicing factors are linked to various endocrine-related cancers, such as breast, ovarian, prostate, pancreatic, and thyroid, stressing RNA biology’s importance in cancer pathogenesis (**Figure 1**).

This growing body of literature reflects the critical role of RNA-centered processes in cancer biology, particularly in endocrine-related malignancies. For example, alternative splicing has been shown to generate isoforms with distinct functions, contributing to the heterogeneity of cancer cells and affecting various cellular processes, including cell proliferation, apoptosis, and metastasis (3). Aberrant mRNA translation and stability further contribute to the malignant phenotype by altering the expression levels of essential regulatory proteins (6, 7). In addition, post-transcriptional modifications, such as RNA methylation, play critical roles in cancer progression by modulating RNA stability and translation efficiency (8). The dysregulation of noncoding RNAs, including miRNAs, lncRNAs, and circRNAs, interferes with gene regulatory networks and contributes to the hallmarks of cancer, such as constant proliferation, evasion of apoptosis and immune system, and metastasis (4, 9, 10).

Moreover, the dysregulation of RNA-centered processes in endocrine-related cancers offers likely prospects for biomarker discovery, improved diagnosis, and targeted therapies. By untangling the intricate roles of RNA molecules in cancer biology, researchers can identify novel diagnostic biomarkers that reflect the molecular signatures of specific cancer subtypes, facilitating more accurate prognostication and treatment stratification. Moreover, targeting aberrant RNA pathways holds promise for developing precision therapies that selectively interrupt oncogenic signaling while sparing normal cellular functions (11).

In conclusion, the rapidly increasing field of RNA biology has significantly developed our understanding of the molecular understanding of endocrine-related cancers. By clarifying the roles of RNA-centered processes in cancer initiation, progression, and metastasis, researchers are poised to uncover novel therapeutic targets and diagnostic biomarkers that could transform the management of endocrine-related malignancies.

## Highlights from research topic contributions

This Research Topic is aimed at highlighting the growing importance of all areas of RNA biology in all aspects of endocrine-related cancers. The research paper contributed by Ha et al. “Aberrant PI3Kδ splice isoform as a potential biomarker and novel therapeutic target for endocrine cancers” revealed that PI3Kδ is overexpressed in endocrine cancer or solid tumors in general. The PI3Kδ-S splice isoform exhibited a more oncogenic activity (compared to PI3Kδ-L) and was expressed in subgroups of all the cancers they had examined, including prostate, breast, pancreatic, colon, and lung cancers. Compared to the full-length PI3Kδ-L, the splice isoform PI3Kδ-S seemed to be exempt from the inhibition by PTEN. SRPIN340, an SRPK1/2 inhibitor, reversed the aberrant splicing and sensitized the advanced endocrine/solid tumors to the PI3Kδ-specific inhibitor, such as Idelalisib. This was the first systematic analysis of the expression profiles of PI3Kδ splice isoforms across different endocrine/solid tumors. In their conclusion, they suggested that the synergistic inhibitory effects of the Idelalisib/SRPIN340 combination may pave a new path for developing novel therapeutics for the Idelalisib-resistant endocrine/solid tumors (and hematologic cancers, in theory) that express PI3Kδ-S (12).

In the contribution by Li et al. “Exploration of epithelial-mesenchymal transition-related lncRNA signature and drug sensitivity in breast cancer”, they constructed a 10-lncRNA risk score signature based on the lncRNAs associated with the Epithelial-Mesenchymal Transition process and assigned breast cancer patients to the high- and low-risk group according to the median risk score. The immune cell infiltration analysis showed that the prognostic risk signature was related to the infiltration of the immune cell subtype. Drug sensitivity analysis proved that EMT-related lncRNA (ERL) signatures could effectively predict the sensitivity of patients to standard chemotherapy drugs in breast cancer patients and provide guidance for chemotherapy drugs for high-risk and low-risk patients. They concluded that their EMT-related lncRNA (ERL) signature and nomogram had excellent prognostic value and could become reliable tools for clinical guidance (13).

In their contribution, Wang et al. in their research paper “Prognostic model based on m6A-associated lncRNAs in esophageal cancer”, constructed a new m6A-related lncRNA prognostic risk model of esophageal cancer (EC), based on three m6A-related lncRNAs: LINC01612, AC025166.1 and AC016876.2, that can predict the prognoses of EC patients. Additionally, the protein-protein interaction and competing endogenous RNA networks were built to determine the underlying biological mechanisms of these lncRNAs. The results of GO and KEGG analyses could also provide insights to confirm the functions of m6A-associated lncRNAs in EC (14).

Liang and Sun, in their contribution to “Prognostic Alternative mRNA Splicing in Adrenocortical Carcinoma,” their data provided a comprehensive bioinformatics analysis of alternative splicing (AS) events in Adrenocortical Carcinoma (ACC) after they constructed well-performed prognostic predictors for risk stratification in ACC. Their study provided biomarkers for disease progression and a potentially rich source of novel therapeutic targets, and this convinced them that these findings could contribute to clinical cancer management as it provided a comprehensive bioinformatics analysis of AS events in ACC, providing biomarkers for disease progression and a potentially rich source of novel therapeutic targets (15).

A study by Wang et al. on “SP1-induced upregulation of lncRNA LINC00659 promotes tumor progression in gastric cancer by regulating miR-370/AQP3 axis” showed that SP1-induced upregulation of LINC00659 promoted gastric cancer (GC) progression by modulating miR-370/AQP3 axis (Graphical Abstract). Furthermore, they showed that the high levels of LINC006591 were associated with poor clinical outcomes in GC patients. Their findings may help to provide new clues for a better understanding of the pathogenesis of GC and explore the feasibility of lncRNAs-directed diagnosis and treatment for GC (16).

## Conclusion

The rapidly increasing field of RNA biology has emerged as a cornerstone in unraveling the molecular complexities of endocrine-related cancers. The comprehensive exploration of RNA-centered processes, from alternative splicing to noncoding RNA regulation, has highlighted the multifaceted roles of RNA molecules in cancer initiation, progression, and metastasis. Notably, research endeavors have identified dysregulated RNA signatures as potential biomarkers for diagnosis and prognostication while also unveiling promising avenues for targeted therapies. Studies such as those elucidating PI3Kδ splice isoforms, EMT-related lncRNA signatures, m6A-associated lncRNA prognostic models, and the impact of lncRNA LINC00659 in gastric cancer exemplify the diverse contributions driving this field forward. As we continue to decode the complex language of RNA in cancer biology, the insights gained hold great promise for revolutionizing the management and treatment of endocrine-related malignancies, ultimately paving the way towards more personalized and effective therapeutic interventions.

## Author contributions

ZD: Writing – original draft, Writing – review & editing. ML: Writing – review & editing. AK: Writing – review & editing.
